# Two years of SARS-CoV-2 genomic surveillance capacity development in Guinea

**DOI:** 10.1038/s41598-026-46736-y

**Published:** 2026-04-01

**Authors:** N’Faly Magassouba, Emanuele Gustani-Buss, Kekoura Ifono, Emily Victoria Nelson, Jacob Camara, Giuditta Annibaldis, Annick Renevey, Julia Hinzmann, Mette Hinrichs, Sarah Ryter, Ehizojie Emua, Saa Lucien Millimono, Eugene Kolie, Moussa Condé, Bakary Sylla, Nourdine Ibrahim, Stephane Mely, Hugo Soubrier, Joëlle Goüy de Bellocq, Beatriz Escudero-Pérez, Laura N. Cuypers, Elodie Moissonnier, Lien de Caluwé, Jonas Müller, Anke Thielebein, Alexandru Tomazatos, Christine Jacobsen, Meike Pahlmann, Beate Becker-Ziaja, Cyril Erameh, Sylvanus Okogbenin, Fara Raymond Koundouno, Youssouf Sidibé, Kaba Keïta, Mamadou Boye Keita, Gianluca Loi, Moke Fundji Jean Marie Kipela, Georges Alfred Ki-Zerbo, Seydou Dia, Philippe Lemey, Stephan Günther, Alimou Camara, Barré Soropogui, Liana Eleni Kafetzopoulou, Sanaba Boumbaly, Sophie Duraffour

**Affiliations:** 1 Centre de Recherche en Virologie, Laboratoire des Fièvres, Hémorragiques Virales de Guinée (CRV-LFHVG), Conakry, Guinea; 2 Université Gamal Abdel Nasser de Conakry, Conakry, Guinea; 3 Department of Microbiology, Immunology and Transplantation, Rega Institute, KU Leuven, Leuven, Belgium; 4 Department of Pediatric Infectious Diseases and Immunology, Wilhelmina Children’s Hospital, University Medical Center Utrecht, Utrecht, The Netherlands; 5 Bernhard Nocht Institute for Tropical Medicine (BNITM), Bernhard Nocht Straße 74, 220359 Hamburg, Germany; 6 German Center for Infection Research (DZIF), partner site Hamburg– Lübeck–Borstel–Riems, Hamburg, Germany; 7 The European Mobile Laboratory (EMLab), BNITM, Hamburg, Germany; 8 Irrua Specialist Teaching Hospital, Irrua, Edo State Nigeria; 9 Laboratoire des Fièvres Hémorragiques Virales de Hôpital Régional de N’Zérékoré, Nzérékoré, Guinea; 10 Institut National de Santé Publique, Conakry, Guinea; 11 The Global Outbreak and Alert Response Network, Geneva, Switzerland; 12 World Health Organization (WHO), Conakry, Guinea; 13 Institute of Vertebrate Biology of the Czech Academy of Sciences, Brno, Czech Republic; 14 Evolutionary Ecology Group, Department of Biology, University of Antwerp, Antwerp, Belgium

**Keywords:** Computational biology and bioinformatics, Diseases, Genetics, Health care, Microbiology

## Abstract

The COVID-19 pandemic significantly accelerated the development of genomic surveillance capabilities worldwide, though equitable access remains a challenge. On 12 March 2020, Guinea, a low-income country in West Africa, reported its first COVID-19 case; however, no local genomic infrastructure was available at the time. A year later, a long-term training program program was initiated to establish a SARS-CoV-2 nanopore sequencing unit at the *Centre de Recherche en Virologie*,* Laboratoire des Fièvres Hémorragiques Virales de Guinée* (CRV-LFHVG) in Conakry, Guinea. Here, we describe the establishment of this capacity and its role in uncovering SARS-CoV-2 circulation dynamics in the region. We established a local hub for comprehensive sequencing training (wet-lab and bioinformatics), where SARS-CoV-2-positive samples, collected as part of routine diagnostic activities from July 2020 to July 2022, were retrospectively and prospectively sequenced using the ONT MinION device. Consensus genomes were generated for variant typing and GISAID-submission. Retrospective phylodynamic analysis was performed. By July 2022, the laboratory had generated 238 SARS-CoV-2 consensus sequences with a median genomic recovery of 98.1% [range: 90.5–99.4], representing 0.64% of the 37,464 confirmed cases reported in the country as of 29 July 2022. These sequences encompassed four waves of infection, with the Delta (21 A, 21I and 21 J) and Omicron (21 K and 21 L) variants of concern (VOCs) accounting for 84% of all identified lineages. Phylogeographic reconstructions revealed introductions of Delta/B.1.617.2 and Delta/AY.37, as well as of Omicron/BA.1.1 and Omicron/BA.1.15.1, potentially from the neighboring Western, Eastern and Middle African regions. Retrospective and prospective sequencing output was > 0.5% of the total positive samples and the results were communicated to the health authorities during the pandemic as in two preliminary variant identification reports, followed by six official reports. This work underscores key findings during a global health crisis and offers operational guidance to support future genomic surveillance initiatives in low- and middle-income countries. Sustained financial investment, dedicated time, specialized expertise, efficient logistics, and local ownership are essential for long-term implementation of such capacities.

**Supplementary Information:**

The online version contains supplementary material available at 10.1038/s41598-026-46736-y.

## Introduction

Since the emergence of the severe acute respiratory syndrome coronavirus 2 (SARS-CoV-2) in 2019, and, as of December 2025 more than 778 million cases of coronavirus disease 2019 (COVID-19) have been diagnosed worldwide causing more than seven million deaths and substantial economic losses^1,2^.

In late 2020, SARS-CoV-2 variants emerged carrying a large number of mutations, raising concerns about their impact on global public health. As a result, these variants were classified into categories designated as Variants of Concern (VOCs), Variants of Interest (VOIs) and Variants under Monitoring (VUMs)^3–5^. The spread of multiple variants around the world highlighted the critical importance of real-time access to genomic surveillance data for tracking variant emergence and guiding public health efforts globally, including the adjustment of molecular tools, treatments, and vaccines^6^. In particular, it allowed for the characterization of mutations potentially driving changes in transmissibility, disease severity, and immune escape^7^. During the pandemic, Alpha (20I; B.1.1.7), Beta (20 H; B.1.351), Gamma (20 J; P.1), Delta (21 A, 21I and 21 J; B.1.617.2 and AY.37), and Omicron (21 K or BA.1 and 21 L or BA.2; B.1.1.529) variants were classified as VOCs^8,9^. A few VOCs were first reported on the African continent including Beta and Omicron in South Africa^10,11^, as well as the VOIs Eta (21D; B.1.525) in Nigeria^12^. The timely detections of Eta and Omicron variants in South Africa prior to their global spread are two of the many examples highlighting the beneficial impact of locally run sequencing capacities in supporting pandemic control efforts^10,11^.

The availability of locally operated diagnostic and genomic surveillance facilities during the COVID-19 pandemic exacerbated global disparities in diagnostic capacity, with investment funds primarily bolstering infrastructure development in high-income countries^13–16^. Specifically, Brito and colleagues showed that 78% of high-income countries sequenced > 0,5% of their COVID-19 cases compared to only 42% of low- and middle-income countries (LMICs) meeting that threshold in the pandemic’s first two years^13^. In line with this, the World Health Organization (WHO) advocated for a ten year strategy to enhance global genomic surveillance, aiming to promptly identify pathogens as part of epidemic and pandemic preparedness^17^. The African continent saw the establishment of multiple local sequencing facilities, improving variant monitoring and enabling the characterization of pandemic waves^16,18–21^. This led to the identification of four successive waves in West Africa between March 2020 and March 2022, with the first wave peaking in July 2020, the second in January-February 2021, the third in August 2021 and the fourth in January 2022, each of them driven by distinct variant circulation dynamics^19^.

Guinea is a low-income West African country and since the first detection of SARS-CoV-2 in the country on 12 March 2020, Guinea has reported a total of 38,582 cases and 468 deaths (1,2% case fatality rate, as of November 2025)^1^. Retrospective sequencing has provided insights into the circulation of variants in Guinea during the two first waves of the pandemic (i.e., 2020 and early 2021) by analyzing 136 and 19 consensus genomes respectively^22,23^. Consistent with the broad West African lineage landscape, lineages 20 A and 20B were predominant in Guinea in 2020, whereas the VOC Alpha (20I) and VOI Eta (21D) were the major lineages present during the second wave in early 2021 ^19,22,23^. Later, recombinant XBB lineage descending from BA.2 variants would be detected in Guinea in the second half of 2022^24^, shortly before the XBB became dominant in the eastern hemisphere^25^. To complement the regional sequencing efforts of Guinea and improve our understanding of in-country variant diversity and dynamics, we established a local nanopore genomic surveillance unit during the pandemic and analyzed the genomic epidemiology of locally generated SARS-CoV-2 genomes. The benefit of such a local setup at the *Centre de Recherche en Virologie*, *Laboratoire des Fièvres Hémorragiques Virales de Guinée* (CRV-LFHVG), Conakry, was previously highlighted during the Ebola virus disease resurgence and the Marburg virus detection in Guinea^26,27^. The feasibility of deploying and implementing nanopore technology has also been detailed in our previous work^28–30^. Here, we underscore the local, regional and global impact of locally operated sequencing platforms and bioinformatic tools in supporting genomic surveillance efforts set up during a public health emergency. This study highlights the challenges faced during in-country implementation and emphasizes the collaborative nature of the work. In addition to providing a crucial operational roadmap for future initiatives aimed at establishing local nanopore sequencing units in resource-limited settings, we use genomic epidemiology to deepen our understanding of SARS-CoV-2 transmission patterns in West-Africa.

## Materials and methods

### Ethical approval and Nagoya permit

This descriptive research, using anonymized diagnostics surveillance data, has been approved by the National Ethics Committee of Guinea (CNERS) under the number 165/CNERS/24. This work is part of the Nagoya permit number 006/2023/PN.

### Definition of the study dataset

Data were included in the study dataset if they were obtained from samples that had been sequenced at CRV-LFHVG and yielded a genomic recovery fitting the threshold for GISAID submission (50%). The samples had various origins: (i) they directly originated from CRV-LFHVG routine diagnostics activities as part of prospective or retrospective sequencing activities (total of 199 samples with GISAID-submitted sequences generated, Supplementary Table S1), or (ii) they originated from other laboratories in Guinea including *Laboratoire des Fièvres Hémorragiques Virales de l’Hôpital Régional de N’Zérékoré* (LFHV-HRNZE, N’Zérékoré) and the *Institut National de Santé Publique* (INSP), which shared their materials for the retrospective sequencing service offered by CRV-LFHVG with a total of 34 RNAs from LFHV-HRNZE and five from INSP. In total, 238 GISAID-submitted sequences have been generated by the CRV-LFHVG laboratory and used for phylogenetic analysis (Table 1).

### Samples, viral RNA extraction and SARS-CoV-2 diagnostics at CRV-LFHVG

Nasopharyngeal swabs collected as part of the in-country surveillance system for COVID-19 were sent to CRV-LFHVG for real-time reverse transcription PCR (RT-PCR) processing as part of routine diagnostic activities. Various kits for the inactivation of samples, extraction of RNA, and RT-PCR were used during the pandemic depending on what was available from donations that were made, kit availability and supply from manufacturers. All kits were used according to manufacturers’ instructions. Extraction kits included (i) DaAn Gene nucleic acid extraction kit (DaAnGene Co Ltd., China), (ii) QIAamp Viral RNA Mini Kit (Qiagen, Germany), (iii) Quick-RNA Viral Kit (Zymo Research, United States) and (iv) AmpliSens^®^ Ribo-prep (InterLabService, Russia). The RT-PCR assays included (i) Fosun COVID-19 RT-PCR detection Kit (Fosun Diagnostics, China), (ii) Novel Coronavirus (2019-nCoV) Nucleic Acid Diagnostic Kit (Sansure Biotech Inc, China), (iii) Detection Kit for 2019-nCoV (PCR-Fluorescence) (DaAnGene Co Ltd., China), (iv) RealStar^®^ SARS-CoV-2 RT-PCR Kit 1.0 (altona Diagnostics, Germany), and (v) AmpliSens^®^ SARS-Coronavirus (InterLabService, Russia). Two real-time thermal cyclers platforms were used including the Rotor Gene Q^®^ (Qiagen, Germany) and BioRad CFX96™ (BioRad, Germany). Leftover RNAs after RT-PCR setup were kept at 4 °C until results were validated according to manufacturers’ instruction algorithms. From September 2021 to July 2022, RNAs with a SARS-CoV-2 positive RT-PCR result were selected and transferred to the sequencing laboratory for further processing with sequencing. The retrospective sequencing activities were performed using stored leftover swab aliquots (-20 °C) which were collected during the period of July 2020 to September 2021. The lack of material stored prior to July 2020 did not allow for an earlier retrospective testing. For the re-testing and sequencing work, RNAs were extracted with the available extraction kits and RT-PCR analysis was re-run with the available assays to verify the cycle threshold (Ct) value before proceeding with sequencing. Overall, all SARS-CoV-2 positive RNAs used for variant identification and phylogeographic inference covered a time interval from July 2020 to July 2022.

### Amplicon preparation, MinION library preparation and next generation nanopore sequencing at CRV-LFHVG

Leftover RNA extracts used for next generation sequencing originated from either prospective activity (i.e. routine diagnostics) or retrospective testing. Samples with a cycle threshold (Ct) < 30 were selected and processed for amplicon-based sequencing using Midnight RT PCR Expansion (EXP-MRT001, Oxford Nanopore Technologies (ONT), United Kingdom). Sequencing experimental setup included: (i) inclusion of the negative RNA extraction controls corresponding to each of the RNA batches to be tested, (ii) inclusion of one sequencing negative control per sequencing run, (iii) doubling the volumes of some steps to minimize pipetting errors, (iv) pooling of RNA batches with a range of maximum five Ct values (e.g., samples from Ct 20 to Ct 25 were pooled in one sequencing run), (v) the pooling of a maximum of 24 samples per flow cell. Briefly, RNA was directly used for cDNA synthesis using reagents of the Midnight RT PCR Expansion and the cDNA generated was used as template for the multiplex PCR in two reaction pools. The resulting amplicons from the two PCR pools were combined and processed as per protocol for barcoding and multiplexing using the Rapid Barcoding Kit (SQK-RBK110.96). For the preparation of less than 11 samples, each sample was prepared in replicates to achieve the library concentration required for sequencing. One unique barcode was used per sample. The pooled, barcoded amplicons were quantified using the Qubit Fluorometer (Thermo Fisher Scientific) aiming for a minimum recovery of 600 to 800 ng in a volume of 11 µl. Libraries were loaded onto the R9.4.1 Flow Cells (FLO-MIN106D, ONT) and sequenced on the Mk1C device (ONT) using the MinKNOW version 21.02.2 with fast5 set to 1000 reads per file and live basecalling disabled. Sequencing runs were stopped after ~ 24 h and fast5 files were transferred on a laptop for basecalling and demultiplexing^26^.

### Bioinformatics, consensus generation, lineage identification, data curation and sharing during the pandemic at CRV-LFHVG

Basecalling and demultiplexing were performed using Guppy version 5.0.16 to obtain fastq files. The following parameters were used: high accuracy basecalling, mid-strand barcodes on, and override minimum mid barcoding score on and set to 50. A modem was configured to ensure optimal internet connection for the analysis of fastq files (using approximately 5 Gb of data). The epi2me™ ARTIC SARS-CoV-2 pipeline (wf-artic workflow) with different versions available at each time of analysis was used for consensus generation from fastq files according to the developers’ instructions. Output consensus files were checked using CLC Main Workbench (Qiagen, Germany) and lineages further identified using the Nextclade desktop app (https://clades.nextstrain.org/*)* and Pangolin^8,31^. A total of 238 consensus sequences have been generated at CRV-LFHVG, curated following GISAID’s algorithm and submitted in different batches to GISAID throughout the pandemic^32^. The related metadata are in the Supplementary File S1.

### Phylogenetic analyses

For the analysis performed here, the 238 genomes were assigned to lineages using Phylogenetic Assignment of Named Global Outbreak Lineages (pangolin; v4.3)^33^ and to clades using Nextclade (v3.8.2)^31^. To provide a global context, high-quality complete genome sequences reported from Guinea and from the six global regions were downloaded from GISAID. For further analysis, global genomes related to the lineage diversity assigned in this study were down-sampled biweekly to cover the time interval explored in this study. A total of 3400 sequences collected between 31 December 2019 and 11 November 2022 were obtained as follows: Africa (*n* = 442), Asia (*n* = 556), Europe (*n* = 657), North America (*n* = 571), South America (*n* = 349) and Oceania (*n* = 212). The whole dataset was assigned using Nextclade 3.8.2 ^31^, and the alignment performed using Nextclade CLI (3.7.1) ^34^. The trimming and checking of data integrity were done using Aliview^35^. For the former VOC Delta, the database creation and formatting followed the same criteria as those described above totaling 4557 genomes sampled between 7 November 2020 and 1 April 2022 comprising Asia (*n* = 180), Eastern Africa (*n* = 1809), Europe (*n* = 209), Middle Africa (*n* = 350), North America (*n* = 212), Northern Africa (*n* = 403), Oceania (*n* = 6), South America (*n* = 5), Southern Africa (*n* = 5), and Western Africa (*n* = 818). A similar method was used for the VOC Omicron resulting in 2885 genomes sampled between 5 November 2021 and 23 August 2022, including Asia (*n* = 187), Central America (*n* = 12), Eastern Africa (*n* = 441), Europe (*n* = 343), Middle Africa (43), North America (*n* = 375), Northern Africa (59), Oceania (*n* = 115), South America (*n* = 172), Southern Africa (*n* = 792), and Western Africa (*n* = 237).

Maximum likelihood phylogenetic trees for these datasets were constructed using IQTREE2 (v.2.3.4) and a General Time-Reversible (GTR) with empirical base frequencies and gamma-distributed rate heterogeneity across four categories, which was selected as the best fitting substitution model^36^. The branch support was inferred by ultrafast bootstrap approximation (UFBoot) and Shimodaira-Hasegawa-like approximate likelihood ratio test (SH-aLRT) with 1000 replicates. A root-to-tip regression was conducted in TempEst v1.5.3 to explore the relationship between genetic divergence and time, evaluating the degree and pattern of temporal signal in the dataset, and to exclude outliers or identify assembly issues^37^.

### Phylogeographic discrete diffusion analysis of selected cluster for the VOCs Delta (AY.37 and B.1.617.2) and Omicron (BA.1.15 and BA.1.1)

A spatiotemporal reconstruction using BEAST v1.10.5 with BEAGLE v4.0.0 was performed using supported subtrees (considering UFBoot and SH-like replicates) clustering with the most frequent lineages identified here to optimize the high-burden computational analysis^38,39^. The substitution model specified was Hasegawa, Kishino, Yano (HKY) nucleotide with gamma-distributed rate variation among sites^40^, combined with a strict molecular clock model (fixed rate = 8 × 10^− 4^ substitutions per site per year), a non-parametric Skygrid coalescent tree model, informed by root height prior distribution^41^. To model the location transition, the asymmetric discrete state diffusion in a Continuous-Time Markov Chain process using the Bayesian stochastic search variable selection (BSSVS) was used to determine routes of significant non-zero migration rates, estimating the posterior inclusion probability (State Posterior Probability or SPP), which provides the supported fluxes by Bayes factor test (Bayes factor > 3) ^42^. Markov jump counts between locations were extracted using the BEAST tool TreeMarkovJumpHistoryAnalyzer^43^. The Markov chain Monte Carlo analyses were run for 200 million steps sampling every 10,000 steps. The effective sample size (ESS) for each parameter was inspected with TRACER v.1.7 and the runs were combined with LogCombiner^38,44^. For each selected cluster (subtree), a maximum clade credibility (MCC) tree was generated from the posterior samples using TreeAnnotator^39^. The resulting trees were visualized and annotated using the *ggtree* package^45^.

## Results

### The context for capacity development

**Fig. 1:**
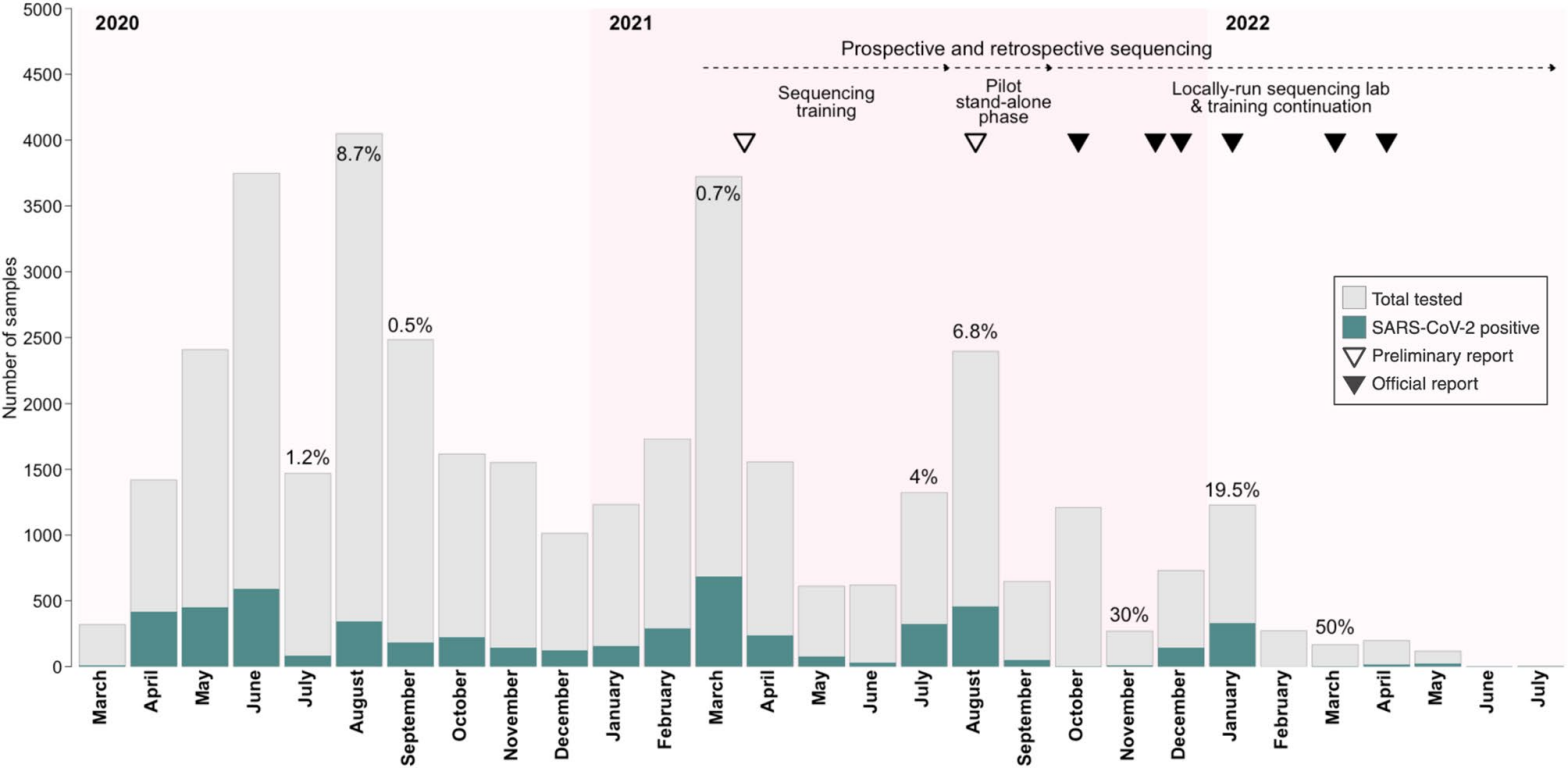
Overview of CRV-LFHVG diagnostics activities and timeline of nanopore sequencing capacity development. The bar plot depicts the data presented in Table S1, indicating the number of samples tested per month for SARS-CoV-2 by RT-PCR. The percentages (%) indicate sequenced positive samples for the month of interest (total of *n* = 199 sequenced from the positive samples reported by CRV-LFHVG only). The timeline indicates the different phases of nanopore sequencing capacity development which started 15 March 2021 and is shown here until July 2022 for ease of visualization, although it continued until the end of 2022. Empty triangles indicate preliminary reports shared with the Ministry of Health based on partial SARS-CoV-2 genomes recovered (range 40–93%) allowing for probable variant identification. Solid black triangles indicate official reports shared with the Ministry of Health after SARS-CoV-2 genome recovery increased to > 90% allowing for variant identification and GISAID data deposition.

As the reference laboratory in Guinea for measles and viral hemorrhagic fevers, including Ebola virus disease and yellow fever, CRV-LFHVG was mandated by the Guinean Ministry of Health to assist with the in-country testing for SARS-CoV-2. Between March 2020 and July 2022, the laboratory tested 38,130 samples, of which 5420 were positive for SARS-CoV-2 (Fig. 1, Supplementary Table S1). Overall, this accounted for approximately 5,3% of the number of tests performed in Guinea during this period (Guinea surveillance data with 725278 tests, 12 March 2020 to 29 July 2022) and led to the diagnosis of about 14,5% of all confirmed cases (Guinea surveillance data with 37464 confirmed cases, 12 March 2020 to 29 July 2022). Furthermore, the establishment of the genomic capacity accounted for 3,7% (*n* = 199) of all positive samples at CRV-LFHVG (*n* = 5420) being sequenced (Supplementary Table S1). The inclusion of sequenced samples from other laboratories, LFHV-HRNZE (*n* = 34) and INSP (*n* = 5), respectively, resulted in a set of 238 SARS-CoV-2 genomes, accounting for 0,64% of all cases confirmed and 22.9% of sequences generated in the country and deposited in GISAID (March 2020 to December 2023). During the peaks of the pandemic, the number of diagnostics tests reached up to 135 tests per day, which were manually performed by lab staff (i.e. no automated platforms). The deployment of in-country sequencing at CRV-LFHVG in 2021 during the Ebola virus disease resurgence ultimately stimulated the launch of the SARS-CoV-2 genomic surveillance capacity development program, following a request from the *Agence de Sécurité Sanitaire* (ANSS) of the Guinean Ministry of Health. The BNITM, through its humanitarian aid cluster, the European Mobile Laboratory (EMLab), and with support from the WHO/GOARN and Guinean health authorities, planned for the implementation of a two-year training program aiming to establish an independently-operated nanopore sequencing unit for SARS-CoV-2 in Guinea.

### Logistical considerations in developing SARS-CoV-2 nanopore sequencing capabilities

Between March 2021 and December 2022, a total of ten missions to Guinea were conducted, with 32 staff deployed (i.e., deployment mechanisms through either GOARN/WHO/EMLab or BNITM/EMLab), to support CRV-LFHVG with diagnostics strengthening and implementation of nanopore sequencing for genomic surveillance. This included scaled-up training encompassing both wet lab and bioinformatics components. Expert teams of three to five staff rotating every four to five weeks from March 2021 to June 2021 were deployed (i.e.17 experts). From August to December 2021, the frequency of training decreased, with three missions of two- to three-weeks each and a total of seven specialized staff deployed. In early, middle and end of 2022, three training missions (three- to four-weeks each) with eight experts took place. Having French-speaking experts on teams was a key component of ensuring efficient communication and knowledge transfer. During this period, six local staff were trained and two became heads of the sequencing unit. The logistic backbone from Germany to Guinea was substantial, consisting of 20 shipments in 2021 and six in 2022, either via courier or as checked luggage of deployed teams. These supplied the laboratory with reagents, consumables and equipment to set up and sustain diagnostics and sequencing operations. Redesigning of the laboratory organization, rewiring of the infrastructure and installation of additional generators were necessary to prevent contamination risks and support access to uninterrupted power. Implementation of standard operating procedures (SOPs) encompassing laboratory, bioinformatics, data storage and back-up processes ensured smooth implementation of a quality assurance management system for delivery of quality data. The supplementary Figure S2 provides an example of a minimum laboratory setup and minimum necessary equipment.

**Fig. 2:**
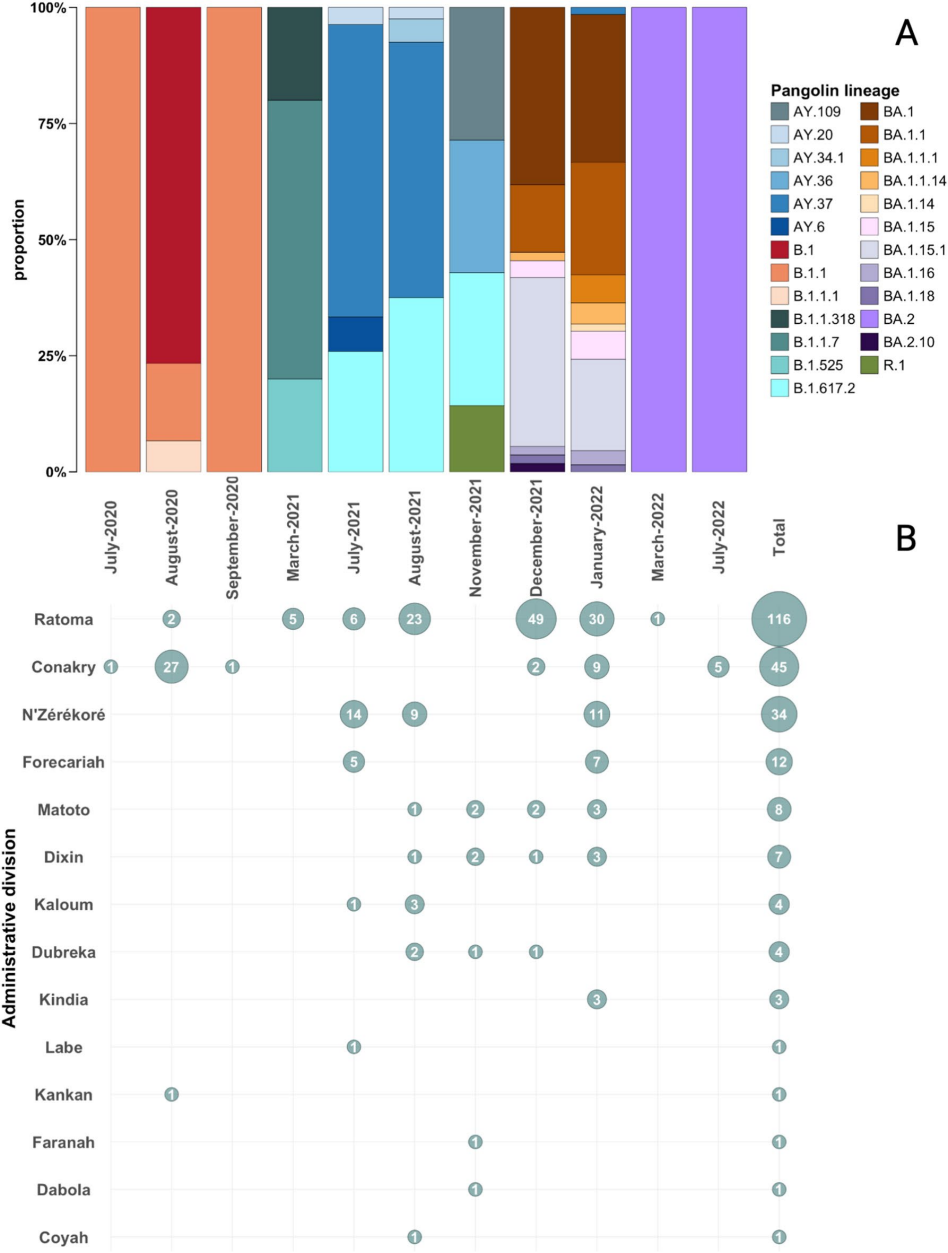
Overview of SARS-CoV-2 genomic surveillance conducted between July 2020 and July 2022. (**A**) Temporal distribution of SARS-CoV-2 lineage diversity identified in the study dataset. (**B**) Sequencing output representing the number of high-coverage (> 90%) SARS-CoV-2 consensus genomes generated, stratified by sample origin.

Between March and August 2021, two amplicon-based sequencing protocols were tested at CRV-LFHVG, namely the ARTIC nCoV-2019 sequencing protocol V3 (ARTIC network and Integrated DNA Technologies GmbH (IDT), Germany) and the NEBNext^®^ ARTIC SARS-CoV-2 Companion Kit (New England Biolabs, United Kingdom). Considering minor optimisations between runs in an attempt to improve genomic recoveries, a total of nine sequencing runs were performed on 57 SARS-CoV-2-positive samples, all of which yielded low genomic recoveries [median of 46,9%, range: 6–93,1%, Inter-Quartile Range (IQR): 25,1–84,9%]. None of these sequences could be submitted to GISAID, as the threshold for submission (50%) was not met. Yet, preliminary findings could still be used for the identification of the VOCs Alpha and Delta, as well as the VOI Eta, which were immediately reported in April and August 2021 to the Ministry of Health of Guinea. From October 2021, the use of the ONT Midnight kit (see Materials and Methods) led to the successful retrieval of 238 consensus sequences with a median genomic recovery of 98,1% [range: 90,5–99,4%, IQR: 96,5–99,3%] (Fig. 2B).

### Impact within and beyond Guinea

Until April 2022, a total of six variant monitoring official reports were shared with the Guinean health authorities before GISAID submission (Fig. 1). The estimated cost for the program between March 2021 and August 2022 was 500,000 €. In 2023 the laboratory was externally evaluated through an international External Quality Assessment scheme SARS-CoV-2 Sequencing-A for accuracy, lineage identification and interpretation, obtaining an overall score of 100%. By October 2021, the SARS-CoV-2 sequencing unit at CRV-LFHVG was fully operational, enabling other laboratories in Guinea to send samples for variant identification. This resulted in additional SARS-CoV-2 sequences from LFHV-HRNZE (*n* = 34) and INSP (*n* = 5). Furthermore, the unit facilitated transborder collaboration with the Irrua Specialist Teaching Hospital (ISTH) in Edo State, Nigeria, which sought to establish similar capabilities. Using CRV-LFHVG as a hub, six Nigerian scientists participated in two separate 2-week sequencing training sessions from late 2021 to the end of 2022, making the Guinean laboratory a regional training hub where technical knowledge was acquired by experienced trainers who would eventually teach others at their original laboratories (i.e. trainers of trainers).

### Lineage characterization, July 2020 to July 2022

A total of 238 sequences were generated by retrospective and prospective sequencing at CRV-LFHVG, covering the four waves of the pandemic, from July 2020 to July 2022 (Figs. 1 and 2B, Supplementary figure S1). The distinction between retrospective and prospective sequencing is dictated here mainly by constraints pertaining to logistics, human resources, sequencer throughput and sample quality. In the context of capacity building during a health crisis, these are likely to affect sequencing and reporting timelines. Here, we made this arbitrary distinction at the 14 days mark between sampling and sequencing dates (i.e., 14 days or more between these timepoints means that sequencing is retrospective). Most of the samples sequenced originated from the Conakry prefecture (75,6%, *n* = 180 samples labeled as Conakry, Ratoma, Kaloum, Dixin, Matoto), followed by N’Zérékoré (14,4%, *n* = 34), Forecariah (5%, *n* = 12) and a minor percentage from various regions throughout the country (5%, *n* = 12). The genomes were assigned to ten clades (Nextclade classification) or 23 lineages with four VOCs and VOI identified as per WHO label including Omicron, Delta, Alpha and Eta (Supplementary Table S2). During the first wave in 2020, the lineages B.1, B.1.1, B.1.1.1 and B.1.1.318 (clades 20 A, 20B and 20D) were predominant, followed by a diversity shift in early 2021 with the appearance of the VOC Alpha B.1.1.7 (20I) and VOI Eta B.1.525 (21D) (Fig. 2). From July 2021 to November 2021, the VOC Delta B.1.617.2, AY.34.1, AY.36, AY.37, AY.6 (21 J and 21 A) was the most identified variant in our dataset. From December 2021 and until July 2022, nearly all sequences were identified as VOI Omicron BA.1, BA.1.1, BA.1.1.1, BA.1.1.14, BA.1.14, BA.1.15.1, BA.1.16, BA.1.18, BA.2 and BA.2.10 (21 K and 21 L, Supplementary Table S2 and Fig. 2A). The observed lineage frequencies may reflect sampling patterns rather than actual national prevalence. The mutation annotation provided by Nextclade showed that the Spike (S) protein had the highest number of amino acid substitutions with 52,5% (*n* = 123) of all analyzed samples, harboring mutations such as S: K417N, S:N440K, S:G446S, S:E484A, S:Q493R, S:G496S, S:Q498R, S:N501Y and S: Y505H, while one mutation, S:L452R, was seen in 31,5% (*n* = 75) of the genomes.

### Global phylodynamic analysis

**Fig. 3:**
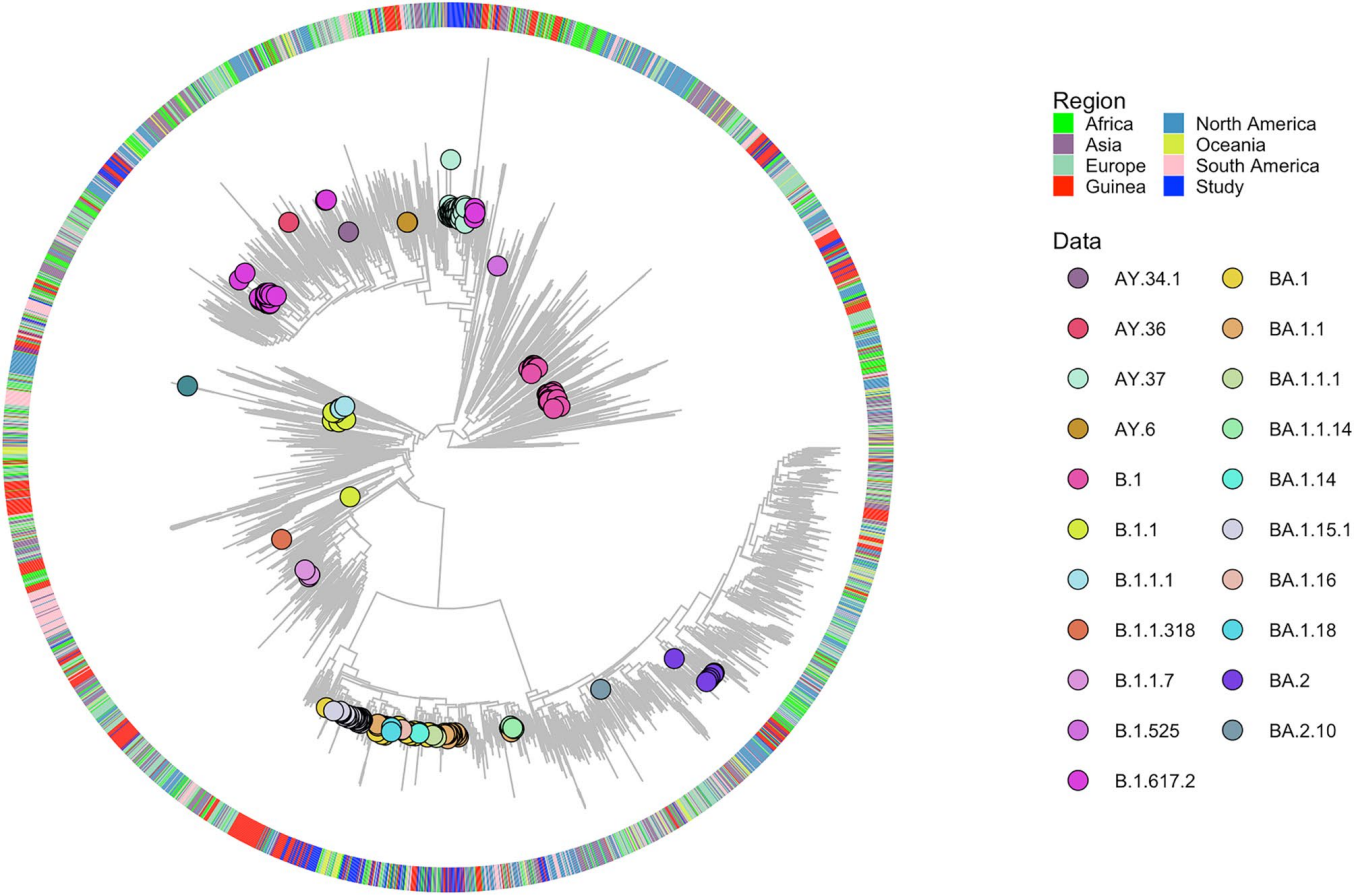
Maximum-likelihood phylogeny and lineages of the 238 sequences generated in this study together with 3400 genomes representative of six global regions. These sequences, along with those from Guinea and the study dataset are identified by the assigned colors in the tree annotation. Lineages identified in our dataset are color-coded on tree branches and identified in the legend.

To place the SARS-CoV-2 genetic diversity identified from our dataset in a global context, we performed a time-calibrated analysis starting with a maximum likelihood phylogenetic tree. A dataset of well-curated, complete and high-quality genomes (*n* = 3400) representing the different clades from six regions worldwide were retrieved from GISAID and added to the 238 sequences generated locally (Fig. 3). The phylogeny shows well-supported subtrees containing the study genomes, suggesting multiple potential introductions of SARS-CoV-2 in Guinea and also fitting the global distribution of SARS-CoV-2 in the respective period of the pandemic. These subtrees consisted in large clusters of the Delta (third wave) and Omicron (fourth wave) VOCs.

**Fig. 4:**
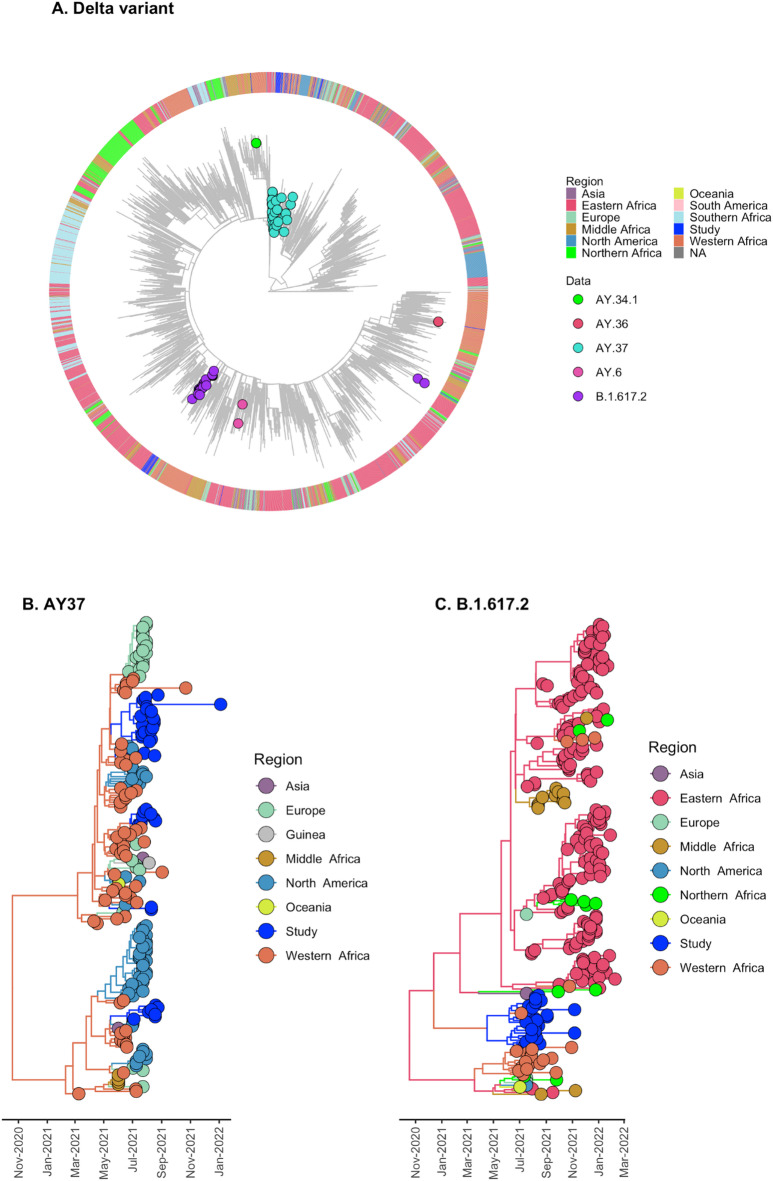
(**A**) Maximum-likelihood phylogeny of the Delta variants for 4557 sequences representing global and regional diversity of variants sampled (colored circles) in Guinea in 2021 and used to identify subtrees clustering the lineages B.1.617.2 and AY.37. (**B**) Bayesian discrete phylogeographic reconstruction (asymmetric discrete state) of genomic clusters, summarized in Maximum clade credibility trees for AY.37 and (C) B.1.617.2. Branch colors represent regions of interest in the reconstruction.

To gain insights into the patterns of introduction and progression within Africa of the two most frequently identified VOCs in this study (Delta and Omicron), we tailored a phylogenetic analysis to examine their diversity and distribution across continental and worldwide representative clades. We then focused on the five subregions of Africa (i.e., Northern, Southern, Eastern, Western and Middle). The VOC Delta was first reported in India in late 2020, and thus 4557 Delta variant sequences were gathered to study the local (Guinea) and global (Africa) context of the dispersion (Fig. 4). The analysis supports two subtrees, one represented by the lineage AY.37 (*n* = 40, 16,8%) (Fig. 4B) and one by B.1.617.2 (*n* = 25, 10,5%) (Fig. 4C). To further explore the introduction and circulation of AY.37 and B.1.617.2, Bayesian discrete phylogeographic analysis was performed to model the spatial transitions between the regions evaluated here and for each subtree. The introduction of the Delta lineage AY.37 was estimated to have originated from West Africa (SPP = 0,99) seeding into Guinea on 25 May 2021 (95% highest posterior density (HPD): 23 May–23 July 2021) (Fig. 4B). For lineage B.1.617.2, the ancestral location reconstruction provided marginal support for Western Africa as a potential source (SPP = 0,39), suggesting considerable uncertainty in the inferred source location. The estimated timing of introduction was 4 April 2021 (95% HPD: 6 March–30 April 2021), although this estimate should be interpreted with caution given the low posterior support for the ancestral state and limited data available (Fig. 4C).

**Fig. 5:**
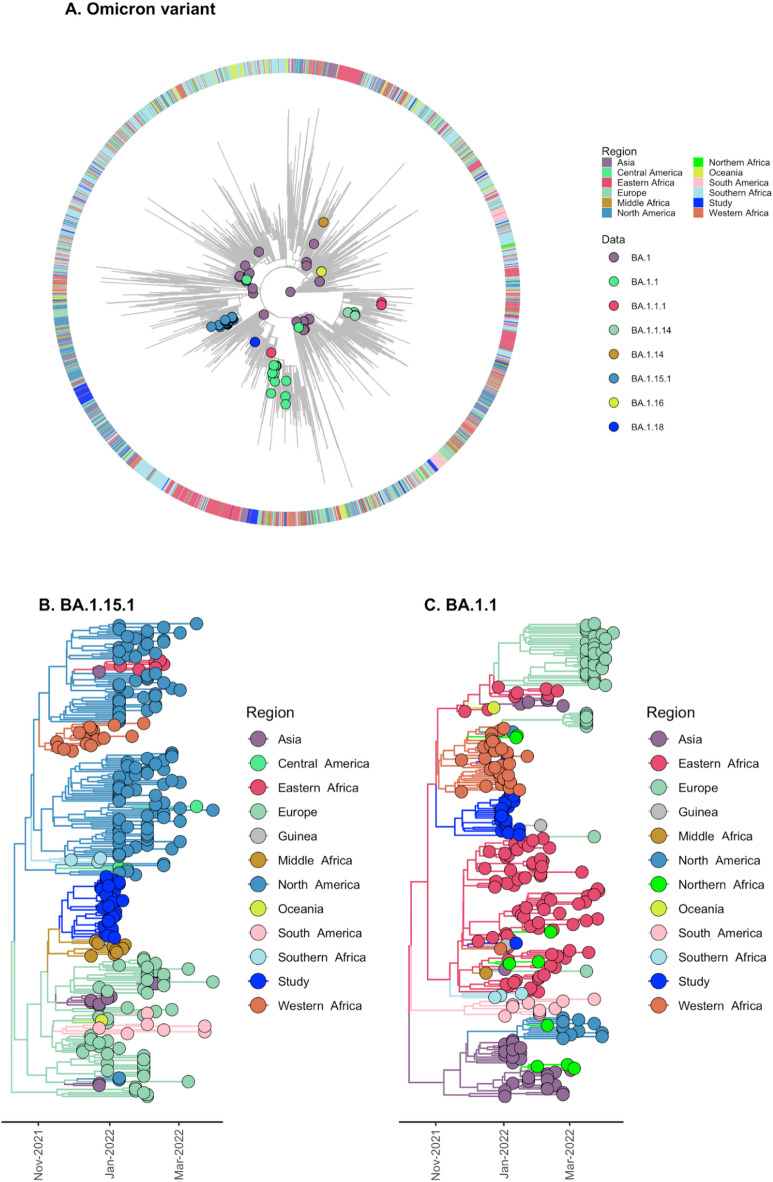
(**A**) Maximum-likelihood phylogeny of Omicron variants for 2885 sequences representing global and regional diversity of variants sampled (colored circles) in Guinea in 2022 and used to identify subtrees clustering the lineages BA.1.15.1 and BA.1.1. (**B**) Bayesian discrete phylogeographic reconstruction (asymmetric discrete state) of genomic clusters, summarized in Maximum clade credibility tree for BA.1.15.1 and for (C) BA.1.1. Branch colors represent regions of interest in the reconstruction.

A similar strategy was used for Omicron which was first detected in South Africa and Botswana in mid-November 2021. A total of 2885 sequences representing all BA.1 lineage and sub-lineages worldwide were assembled to produce a phylogenetic tree (Fig. 5). Our analysis revealed multiple introductions for BA.1 and two supported subtrees for lineages BA.1.15.1 (*n* = 38, 16%) and BA.1.1 (*n* = 27, 11,3%) (Fig. 5A). The phylogeographic inference for BA.1.15.1 dated the introduction into Guinea on 23 November 2021 (95% HDP: 14 November – 30 November 2021) with a likely source from Middle Africa (SPP = 1) (Fig. 5B). For the BA.1.1 variant, phylogeographic analysis inferred Eastern Africa as the most likely source (SPP = 1), with an estimated introduction date of 24 November 2021 (95% HPD: 10 November – 6 December 2021) (Fig. 5C). However, this inference is conditional on the completeness and representativeness of sequences available in GISAID, whose uneven regional sampling may bias source attribution. Accordingly, while Eastern Africa received the highest statistical support, alternative origins cannot be excluded.

## Discussion

Since SARS-CoV-2 was first identified, genomic surveillance combined with evolutionary epidemiology has helped untangle the complexity of worldwide variant spread. However, disparities in genomic surveillance capabilities among LMICs relative to high-income countries have been clearly highlighted^13^. The successful establishment of genomic surveillance capacity led to the generation of 238 consensus sequences obtained locally by prospective and retrospective sequencing of diagnostic samples collected between July 2020 and July 2022. The generated genomic dataset positions Guinea slightly above the sequencing intensity threshold of 0,5% suggested by Brito and colleagues as a potential marker for genomic sequencing ability although a turnaround time of < 21 days from sample collection to GISAID submission was also suggested as a proxy for genomic surveillance capability^13^. The median of 172,5 days [range: 37–563; IQR: 116–317] (see Supplementary Figure S1) observed here is likely biased by a combination of factors such as late implementation during the pandemic (October 2021), substantial yield of retrospective sequencing, time allocation for training, data curation and sharing to health authorities prior to GISAID submission. Such bottlenecks should definitely be considered in improving future preparedness activities for timely sharing. The genomic data was provided to the Guinean Ministry of Health (MOH) in the form of eight variant reports (including two preliminary ones). These reports provided a basis for public communication^46–48^, focusing on raising population awareness of variants circulating in the country and advocated for adherence to protective measures (e.g., masks and vaccination)^46^. Whilst the reports contributed to situational awareness and genomic surveillance of the COVID-19 pandemic in Guinea, to our knowledge and within the context of our work, these contributions remain descriptive, gathered from press releases and expert panel discussions.

The lineage distribution is similar with previously reported dynamics during the early waves in Guinea and West-Africa^19,22,23^. Initially dominated by 20 A and 20B clades, the Alpha VOCs and the Eta VOI subsequently dominated the epidemiological landscape during the second wave in Guinea. Lineages Delta and Omicron appeared to dominate the third and fourth waves in Guinea with a predominance of Delta/AY.37 and Omicron/BA.2, which had not yet been reported in Guinea at the time. Our results may be biased by sampling limited to certain districts, sample selection based on low Ct values and timeline of operational readiness affecting overall lineage frequencies, thus leading to the VOCs Delta and Omicron dominating the dataset. Yet, identification of virus lineages and understanding their diffusion across time allows for mutation screening to identify potential sites located primarily in the receptor binding domain (RBD) of the viral Spike protein which are linked with immune escape and reduced vaccine efficacy^7^. Thus, amino acid changes identified in our dataset within the RBD, N501Y, P681H, K417T, E484K, L452R, T478K have been reported to contribute to a differential binding affinity to the ACE2 receptor and alter pathogenicity, immunogenicity and immune escape^49^.

Our initiative provided the opportunity to explore the introduction dynamics of the most frequent VOCs in Guinea, namely Delta/B.1.617.2 and Delta/AY.37, as well as Omicron/BA.1.1 and Omicron/BA.1.15. Both were identified as prominent lineages during the second and third waves, which coincidently corresponded to a period of low vaccination coverage (4%) in the country^50^. Our findings suggest that both Delta/B.1.617.2 and Delta/AY.37 were potentially introduced into Guinea from West-African countries, with introduction timelines compatible with their respective estimated global circulation. However, these findings should be interpreted with caution, considering that phylogeographic inference is sensitive to the underlying genomic representation and uneven sampling across regions and time periods, i.e. uneven sampling at regional and international scales and the ensuing bias in public databases. The Delta/B.1.617.2 emerged in October 2020 in India and several sub-lineages circulated prior to its global expansion by March^14,21,51,52^. While constrained by regional sequence availability, our analyses indicated East and Central Africa as probable sources of transmission of Omicron/BA.1.1 and Omicron/BA.1.15.1 into Guinea, likely occurring within a few weeks of their estimated emergence in South Africa in November 2021^10,21,53^. This is in line with previous studies exploring the regional and global spreading of Omicron/BA.1’s reaching more than 30 countries by early December 2021 and further driving global transmission, coinciding with the global relaxation of non-pharmaceutical interventions and absent or low vaccination coverage^19,54–56^. Lockdown or other restrictive measures in Guinea have been previously described and support variant dynamics^22,50^, as well as global mobility in rapidly spreading variants^56^. Global and regional epidemic patterns may vary due to regional particularities, such as restrictions of mobility, waning of immunity, lineage competition and recombination, or high transmission rate, all of which facilitate the emergence of new variants and justify the need to have such genomic capacities in country^21,57^. Omicron thus rapidly displaced Delta on the global scale which was also facilitated by its enhanced immune evasion in the context of vaccination, convalescent sera and antibody therapy^58^. Our findings align with the reported enhanced transmissibility of Omicron, as compared to other variants circulating at the time, as our estimates indicate its introduction in Guinea within a few weeks after its first detection in the middle of November^10,58^.

### Limitations

The results obtained during the establishment and operation of the lab contain spatial and epidemiological bias, due to several technical and operational constraints. The reduced capacity for diagnostic and sequencing possessed by Guinea during the pandemic meant that most of the samples reaching CRV-LFHVG originated from the Conakry administrative region. Logistical constraints related to sample exportation from Guinea for sequencing, combined with limited data on variant circulation during the early stages of the pandemic, may have introduced temporal gaps or restrictions on the selection of samples for sequencing. However, our results mirror the pattern of testing and sequencing observed at national level, where the large majority (874 of 1038, 84,2%) of sequences were generated in the capital region^59^, while the number of SARS-CoV-2 genomes generated from the other regions and submitted to GISAID by December 2023 was as low as 1 (Faranah region) [range 1–46]. The cause is most likely due to the limited diagnostic capability and absence of sequencing capacity outside Conakry, where diagnostic and surveillance were established at the beginning of the pandemic. A similar pattern could be observed for anti-COVID-19 immunisation. Vaccination was effectively conducted in Guinea within the frame of five campaigns between March 2021 and March 2022 ^60^. Vaccination rate was low (4%) in the first months of national implementation efforts and remained so until the emergence of SARS-CoV-2 variants. The immunisation coverage towards the end of 2021 still showed a large difference between Conakry and other regions of Guinea^50^, similar to the pattern of genomic data generated for surveillance. Another aspect with impact on the results relates to sample selection according to Ct values obtained from the diagnostic tests. We have sequenced only samples with Ct < 30 in order to maximize genome recovery, phylogenetic signal and variant identification. Thus, useful information could have been missed when using such a proxy for viral genetic material abundance. As such, to the extent that the proportion of sequenced samples is significant, the results may be representative for the capital region, but not for the entire country. In the context of global spatiotemporal analysis, findings should be interpreted in light of the heterogeneous genomic surveillance data sourced from public databases, as well as the down-sampling strategies employed to curate the dataset and extract subtrees. While these approaches were necessary to ensure computational feasibility and comprehensive representation of global viral diversity, some transmission pathways may remain underrepresented or undetected. Despite providing useful information about VOC circulation in the country, the measurable impact of genomic surveillance on public health policies was not evaluated. Such assessment should be considered in future interventions, while maintaining local sequencing capacity. These measures will increase public health impact and ensure timely response to future outbreaks.

### Operational lessons

Our study offers useful practical lessons learned during a long-term capacity development program, including a model of genomic surveillance for tracking the spatio-temporal dynamics of pathogen variants during an ongoing public health crisis. Previous expertise of the local laboratory workforce in diagnostics and molecular biology has facilitated the successful setup and provided access to good quality genetic material for sequencing. Notably, some aspects should be considered for sustained impact, including troubleshooting training, access to English courses (if applicable), implementation of robust workflows with a quality assurance management system for laboratory process tracking, data validation and curation for high quality data. Furthermore, delivery of broad bioinformatic training ranging from computer programming courses, to computer setup, data analysis, data storage and backup was key to local workforce development and sustainable knowledge transfer. Development of bioinformatic capacity was generally slower, and not at the same pace as the sequencing progress. During our initiative in Guinea, the bioinformatic software used in the lab was ready-to-use (see Materials and Methods), relying on a graphic user interface for ease of access. Bioinformatic capacity was developed in tandem with sequencing capacity, seeking to harmonise the two components and making infrastructure maintenance part of the training. These aspects were implemented by the large number of experts deployed to support diagnostics refreshers, sequencing trainings and knowledge transfer. The online availability of user-friendly tools, such as Nextstrain or Pangolin^8,31,32,34,61^, significantly facilitated SARS-CoV-2 sequencing training with data analysis, variant identification and phylogenetic investigations^57,62^. Yet, basic knowledge of bioinformatics still remains a prerequisite for staff and the scarcity of staff with such expertise remains a major obstacle to maintaining capabilities. Mobilization of funds to foster bioinformatics capacity in LMICs should become a priority^13,17,21,63^. Operational challenges in such settings are considerable, ranging from import-export procedures, security and political context, laboratory location and infrastructure, to chosen sequencing technology, access to the power grid and running water, data access and dissemination rights, or availability to in-country or within-continent supply chains (temperature-controlled) for reagents. These aspects must be addressed to not only build robust genomic surveillance capabilities but also to strengthen resilient and self-sufficient public health systems in such settings^64,65^. The 26 shipments from Germany to Guinea conducted for this study are just one example of such challenges and underline the urgent need for equitable market access, as well as country leadership and engagement in promoting such access^17,21,63–65^.

### Implications for genomic surveillance in low-resource settings

Another highlight of our initiative was the fruitful South-South cooperation with Nigeria, which expedited the establishment of a similar capacity at ISTH in April 2022. Guinea as a training hub was opportune for improving on-site knowledge transfer, best practices and staff expertise in operating such a laboratory in an environment which best reflects the daily reality in LMICs. The cooperation showcased the benefits of establishing a regional training hub where African scientists were trained for later dissemination of acquired knowledge in their original labs, thus making this capacity building model a sustainable one.

Our work shows that setting up a locally-run sequencing hub during a health emergency is feasible with robust financial support, staff dedication, substantial logistics and long-term partnership. Although most of the sequencing was retrospective, the established local sequencing capacity provided the Guinean MoH with useful information about variant circulation, and also stimulated South-South collaboration where disparities in diagnostic and sequencing capacity are still large. Since then, the sequencing remains operational CRV-LFHVG in Conakry, where the local team is currently conducting sequencing autonomously. Activities are ongoing to further enhance genomic surveillance preparedness in Guinea in the event of future epidemics and pandemics. We wish our work to stimulate similar future initiatives in building resilient laboratory systems and advocate for sustained investments.

## Supplementary Information

Below is the link to the electronic supplementary material.

Supplementary Material 1

Supplementary Material 2

## Data Availability

The datasets used and/or analyzed during the current study are available from the corresponding author on reasonable request.

## Abbreviations

AI: Artificial Intelligence
ANSS: Agence Nationale de Sécurité Sanitaire
BNITM: Bernhard-Nocht-Institute for Tropical Medicine
BSSVS: Bayesian Stochastic Search Variable Selection
cDNA: Complementary DNA
CNERS: Comité National d’Ethique pour la Recherche en Santé (National Ethics Committee of Guinea)
COVID-19: Corona Virus Disease 2019
CRV-LFHVG: “Centre de Recherche en Virologie, Laboratoire des Fièvres Hémorragiques Virales de Guinée”
Ct: Cycle threshold
DZIF: Deutsches Zentrum für Infektionsforschung (German Center for Infection Research)
EMLab: European Mobile Laboratory
ESS: Effective Sample Size
Gb: Gigabit
GISAID: Global Initiative on Sharing All Influenza Data
GOARN: Global Outbreak Alert and Response Network
HKY: Hasegawa, Kishino, Yano
INSP: Institut National de Santé Publique
ISTH: Irrua Specialist Teaching Hospital
IQR: Inter-Quartile Range
LFHV-GKD: Laboratoire des Fièvres Hémorragiques Virales de Gueckédou
LFVH-HRNZE: Laboratoire des Fièvres Hémorragiques Virales de l’Hôpital Régional de N’Zérékoré
LMIC: Low- and middle-income countries
MCC: Maximum Clade Credibility
µl: Micro Liter
n: Sample Size
ng: Nano Gram
ONT: Oxford Nanopore Technologies
RBD: Receptor Binding Domain
PCR: Polymerase Chain Reaction
RNA: Ribonucleic Acid
RT-PCR: Real time reverse transcription polymerase chain reaction
S: Spike
SARS-CoV-2: Severe Acute Respiratory Syndrome Coronavirus 2
SH-aLRT: Shimodaira-Hasegawa-like Approximate Likelihood Ratio Test
SOPs: Standard Operating Procedures
SPP: State Posterior Probability
UFBoot: Ultrafast Bootstrap Approximation
VOCs: Variants of Concern
VOIs: Variants of Interest
VUMs: Variants under Monitoring
WHO: World Health Organization
