# Supplementary material for: Two years of SARS-CoV-2 genomic surveillance capacity development in Guinea
Source: Sci Rep. 2026 Apr 1;16:11225. doi: 10.1038/s41598-026-46736-y (PMC13046842; doi:10.1038/s41598-026-46736-y)
Supplement: Supplementary file 1 — Supplementary Material 1 [file 41598_2026_46736_MOESM1_ESM.docx]

**Supplemental material**

Two years of genomic surveillance capacity development in Guinea: an operational roadmap for local implementation in low-income countries and tracking of SARS-CoV-2 circulation dynamics

^*^ Corresponding author: Christine Jacobsen

Bernhard Nocht Institute for Tropical Medicine (BNITM),

Bernhard Nocht Straße 74, 220359, Hamburg, Germany;

[christine.jacobsen@bnitm.de](mailto:christine.jacobsen@bnitm.de),

phone: +49 40 285380 932

Table of Contents

[***Table S1 2***](#_heading=h.feb79f17agjd)

[***Table S2 3***](#_heading=h.1oozt1imqqvi)

[***Figure S1 4***](#_heading=h.xg90jdise6bg)

[***File S1 5***](#_heading=h.wy7gy7xba3l5)

# Table S1. Overview of SARS-CoV-2 testing and number of GISAID-related genome sequences originating from CRV-LFHVG, March 2020 to July 2022. The table shows the amount and proportion of samples tested and sequenced retrospectively and as part of routine diagnostic at CRV-LFHVG in each month between March 2020 and July 2022.

| **Year** | **Month** | **Number of samples tested** | **Number of samples SARS-CoV-2 positive** | **Percentage positive samples (%)** | **Number of samples sequenced and submitted to GISAID** | **Percentage of samples sequenced and submitted to GISAID** |
| --- | --- | --- | --- | --- | --- | --- |
|  |  |  |  |  |  |  |
| **2020** | March | 320 | 10 | 3,1 | 0 | 0 |
|  | April | 1420 | 420 | 29,6 | 0 | 0 |
|  | May | 2408 | 450 | 18,7 | 0 | 0 |
|  | June | 3749 | 591 | 15,8 | 0 | 0 |
|  | July | 1470 | 86 | 5,9 | 1 | 1,2 |
|  | August | 4051 | 345 | 8,5 | 30 | 8,7 |
|  | September | 2483 | 184 | 7,4 | 1 | 0,5 |
|  | October | 1617 | 221 | 13,7 | 0 | 0 |
|  | November | 1553 | 145 | 9,3 | 0 | 0 |
|  | December | 1014 | 126 | 12,4 | 0 | 0 |
|  | *Sub-total* | *20085* | *2578* | *12,8* | *32* | *1,2* |
|  |  |  |  |  |  |  |
| **2021** | January | 1233 | 154 | 12,5 | 0 | 0 |
|  | February | 1732 | 291 | 16,8 | 0 | 0 |
|  | March | 3723 | 688 | 18,5 | 5 | 0,7 |
|  | April | 1556 | 235 | 15,1 | 0 | 0 |
|  | May | 612 | 78 | 12,7 | 0 | 0 |
|  | June | 620 | 32 | 5,2 | 0 | 0 |
|  | July | 1324 | 323 | 24,4 | 13 | 4 |
|  | August | 2395 | 458 | 19,1 | 31 | 6,8 |
|  | September | 649 | 53 | 8,2 | 0 | 0 |
|  | October | 1209 | 1 | 0,1 | 0 | 0 |
|  | November | 270 | 10 | 3,7 | 3 | 30 |
|  | December | 731 | 144 | 19,7 | 49 | 34 |
|  | *Total* | *16054* | *2467* | *15,4* | *101* | *4,1* |
|  |  |  |  |  |  |  |
| **2022** | January | 1229 | 333 | 27,1 | 65 | 19 |
|  | February | 273 | 0 | 0 | 0 | 0 |
|  | March | 167 | 2 | 1,2 | 1 | 50 |
|  | April | 199 | 16 | 8 | 0 | 0 |
|  | May | 118 | 22 | 18,6 | 0 | 0 |
|  | June | 1 | 1 | 100 | 0 | 0 |
|  | July | 4 | 1 | 25 | 0 | 0 |
|  | *Total* | *1991* | *375* | *18,8* | *66* | *17,6* |
|  |  |  |  |  |  |  |
| **2020-2022** | **Total** | **38130** | **5420** | **14,2** | **199** | **3,7** |

# Table S2. Study dataset lineages and frequency. The table shows the amount, proportion and lineage classification of SARS-CoV-2 genomes generated at CRV-LFHVG and used in the genomic epidemiological analysis.

| **Lineage** | **Nextclade** | **WHO label*** | **Number of sequences** | **Percentage** |
| --- | --- | --- | --- | --- |
| B.1 | 20A |  | 23 | 9,7 |
| B.1.1 | 20B |  | 6 | 2,5 |
| B.1.1.1 | 20D |  | 3 | 1,3 |
| B.1.1.318 | 20B |  | 1 | 0,4 |
| B.1.1.7 | 20I | Alpha | 3 | 1,3 |
| B.1.525 | 21D | Eta | 1 | 0,4 |
| B.1.617.2 | 21A | Delta | 3 | 1,3 |
| B.1.617.2 | 21J | Delta | 25 | 10,5 |
| AY.6 | 21J | Delta | 2 | 0,8 |
| AY.34.1 | 21J | Delta | 2 | 0,8 |
| AY.36 | 21J | Delta | 2 | 0,8 |
| AY.37 | 21I | Delta | 40 | 16,8 |
| BA.1 | 21K | Omicron | 41 | 17,2 |
| BA.1.1 | 21K | Omicron | 27 | 11,3 |
| BA.1.1.1 | 21K | Omicron | 3 | 1,3 |
| BA.1.1.14 | 21K | Omicron | 4 | 1,7 |
| BA.1.14 | 21K | Omicron | 1 | 0,4 |
| BA.1.15.1 | 21K | Omicron | 38 | 16,0 |
| BA.1.16 | 21K | Omicron | 3 | 1,3 |
| BA.1.18 | 21K | Omicron | 2 | 0,8 |
| BA.2 | 21L | Omicron | 6 | 2,5 |
| BA.2.10 | 21L | Omicron | 1 | 0,4 |
| R.1 | 20B |  | 1 | 0,4 |
| Total | NA | NA | 238 | 100 |

*The VOCs are Alpha, Delta, and Omicron; the VOI is Eta; the decreasing proportions were as follows: the 21K and 21L (Omicron) represent 53% (n = 126) of the dataset; the 21A, 21I and 21J (Delta) 31% (n = 74); 20A 9,7% (n = 23); 20B 3,3 % (n = 8); 20D 1,3% (n = 3); 21I (Alpha) 1,3% (n = 3) and 21D (Eta) 0,4% (n = 1). NA, not applicable

# Figure S1

**Example of nanopore sequencing laboratory minimal setup.**

Schematic representation of a SARS-CoV-2 nanopore next generation sequencing laboratory for setup in remote settings, as in CRV-LFHVG.

Sample preparation is performed on a clean bench where a basic pipette set, centrifuge and vortex are available. The reaction mastermix is prepared on a separate clean bench and requires a smaller centrifuge, as well as a basic pipette set. For preparation of sequencing libraries, the work is divided between pre- and post-amplification phases, both of which are conducted on separate clean benches. Each bench is equipped with complete pipette sets (10-20 µl, 100-200 µl and 1000 µl), centrifuges (small and medium for the pre-amplification steps) and dry bath. The post-amplification phase requires a magnetic rack for sample clean-up. A separate table is necessary for sequencing and data analysis, together with backup devices in duplicate. All lab equipment is powered by an UPS, separately from the computational unit which has its own power supply. The cold chain ensuring integrity of samples, reagents and nanopore flow cells comprises a 4°C (4 to 8°C) unit and a -20°C (-25 to -15°C). To overcome troubleshooting and sustain uninterrupted activities, every piece of laboratory or sequencing equipment is available at least in duplicate. Created in BioRender. Duraffour, S. (2025)

**
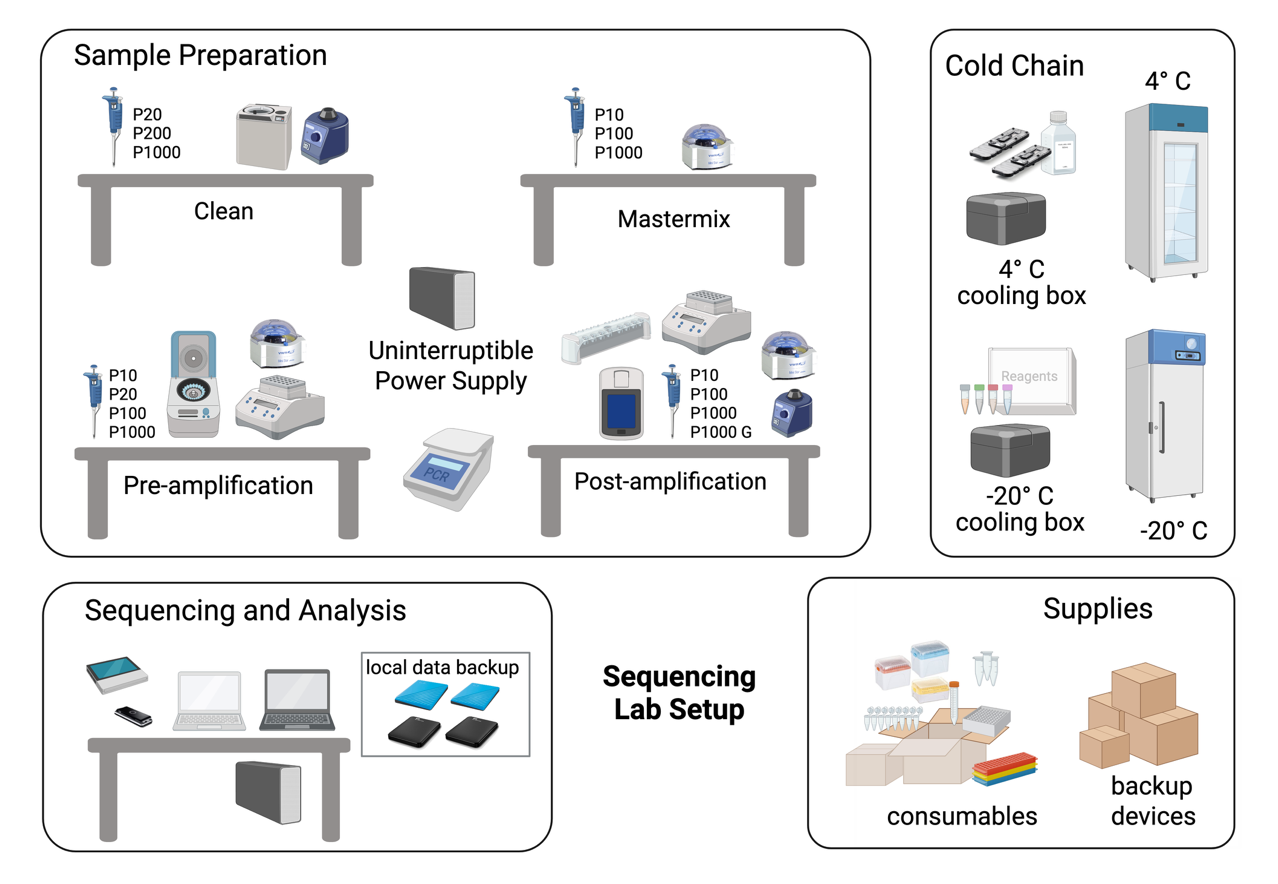
**

# File S1

**Metadata**

**Data Availability**

GISAID Identifier: EPI_SET_250318ve

DOI: https://doi.org/10.55876/gis8.250318ve

All genome sequences and associated metadata in this dataset are published in GISAID’s EpiCoV database. To view the contributors of each individual sequence with details such as accession number, Virus name, Collection, date, Originating Lab and Submitting Lab and the list of Authors, visit 10.55876/gis8.250107tk

**Data Snapshot**

EPI_SET_250318ve is composed of 10,621 individual genome sequences.

The collection dates range from 2019-12-31 to 2022-11-08;

Data were collected in 176 countries and territories.
