# Supplementary material for: Two years of SARS-CoV-2 genomic surveillance capacity development in Guinea
Source: Sci Rep. 2026 Apr 1;16:11225. doi: 10.1038/s41598-026-46736-y (PMC13046842; doi:10.1038/s41598-026-46736-y)
Supplement: Supplementary file 2 — Supplementary Material 2 [file 41598_2026_46736_MOESM2_ESM.docx]

**Supplementary material**

Two years of genomic surveillance capacity development in Guinea: an operational roadmap for local implementation in low-income countries and tracking of SARS-CoV-2 circulation dynamics

^*^ corresponding author: Christine Jacobsen, Bernhard Nocht Institute for Tropical Medicine (BNITM), Bernhard Nocht Straße 74, 220359, Hamburg, Germany; [christine.jacobsen@bnitm.de](mailto:christine.jacobsen@bnitm.de), phone: +49 40 285380 932

Table of Contents

[***Table S1 2***](#_heading=h.feb79f17agjd)

[***Table S2 3***](#_heading=h.1oozt1imqqvi)

[***Figure S1 4***](#_heading=h.xg90jdise6bg)

***Figure S2 5***

[***File S1***](#_heading=h.wy7gy7xba3l5) **6**

# Table S1

**Overview of SARS-CoV-2 testing and number of GISAID-related genome sequences originating from CRV-LFHVG, March 2020 to July 2022.**

| **Year** | **Month** | **Number of samples tested** | **Number of samples SARS-CoV-2 positive** | **Percentage positive samples (%)** | **Number of samples sequenced and submitted to GISAID** | **Percentage of samples sequenced and submitted to GISAID** |
| --- | --- | --- | --- | --- | --- | --- |
|  |  |  |  |  |  |  |
| **2020** | March | 320 | 10 | 3,1 | 0 | 0 |
|  | April | 1420 | 420 | 29,6 | 0 | 0 |
|  | May | 2408 | 450 | 18,7 | 0 | 0 |
|  | June | 3749 | 591 | 15,8 | 0 | 0 |
|  | July | 1470 | 86 | 5,9 | 1 | 1,2 |
|  | August | 4051 | 345 | 8,5 | 30 | 8,7 |
|  | September | 2483 | 184 | 7,4 | 1 | 0,5 |
|  | October | 1617 | 221 | 13,7 | 0 | 0 |
|  | November | 1553 | 145 | 9,3 | 0 | 0 |
|  | December | 1014 | 126 | 12,4 | 0 | 0 |
|  | *Sub-total* | *20085* | *2578* | *12,8* | *32* | *1,2* |
|  |  |  |  |  |  |  |
| **2021** | January | 1233 | 154 | 12,5 | 0 | 0 |
|  | February | 1732 | 291 | 16,8 | 0 | 0 |
|  | March | 3723 | 688 | 18,5 | 5 | 0,7 |
|  | April | 1556 | 235 | 15,1 | 0 | 0 |
|  | May | 612 | 78 | 12,7 | 0 | 0 |
|  | June | 620 | 32 | 5,2 | 0 | 0 |
|  | July | 1324 | 323 | 24,4 | 13 | 4 |
|  | August | 2395 | 458 | 19,1 | 31 | 6,8 |
|  | September | 649 | 53 | 8,2 | 0 | 0 |
|  | October | 1209 | 1 | 0,1 | 0 | 0 |
|  | November | 270 | 10 | 3,7 | 3 | 30 |
|  | December | 731 | 144 | 19,7 | 49 | 34 |
|  | *Total* | *16054* | *2467* | *15,4* | *101* | *4,1* |
|  |  |  |  |  |  |  |
| **2022** | January | 1229 | 333 | 27,1 | 65 | 19 |
|  | February | 273 | 0 | 0 | 0 | 0 |
|  | March | 167 | 2 | 1,2 | 1 | 50 |
|  | April | 199 | 16 | 8 | 0 | 0 |
|  | May | 118 | 22 | 18,6 | 0 | 0 |
|  | June | 1 | 1 | 100 | 0 | 0 |
|  | July | 4 | 1 | 25 | 0 | 0 |
|  | *Total* | *1991* | *375* | *18,8* | *66* | *17,6* |
|  |  |  |  |  |  |  |
| **2020-2022** | **Total** | **38130** | **5420** | **14,2** | **199** | **3,7** |

See **Figure 1** in **Results** for additional information.

# Table S2

**Study dataset lineages and frequency**

| **Lineage** | **Nextclade** | **WHO label*** | **Number of sequences** | **Percentage** |
| --- | --- | --- | --- | --- |
| B.1 | 20A |  | 23 | 9,7 |
| B.1.1 | 20B |  | 6 | 2,5 |
| B.1.1.1 | 20D |  | 3 | 1,3 |
| B.1.1.318 | 20B |  | 1 | 0,4 |
| B.1.1.7 | 20I | Alpha | 3 | 1,3 |
| B.1.525 | 21D | Eta | 1 | 0,4 |
| B.1.617.2 | 21A | Delta | 3 | 1,3 |
| B.1.617.2 | 21J | Delta | 25 | 10,5 |
| AY.6 | 21J | Delta | 2 | 0,8 |
| AY.34.1 | 21J | Delta | 2 | 0,8 |
| AY.36 | 21J | Delta | 2 | 0,8 |
| AY.37 | 21I | Delta | 40 | 16,8 |
| BA.1 | 21K | Omicron | 41 | 17,2 |
| BA.1.1 | 21K | Omicron | 27 | 11,3 |
| BA.1.1.1 | 21K | Omicron | 3 | 1,3 |
| BA.1.1.14 | 21K | Omicron | 4 | 1,7 |
| BA.1.14 | 21K | Omicron | 1 | 0,4 |
| BA.1.15.1 | 21K | Omicron | 38 | 16,0 |
| BA.1.16 | 21K | Omicron | 3 | 1,3 |
| BA.1.18 | 21K | Omicron | 2 | 0,8 |
| BA.2 | 21L | Omicron | 6 | 2,5 |
| BA.2.10 | 21L | Omicron | 1 | 0,4 |
| R.1 | 20B |  | 1 | 0,4 |
| Total | NA | NA | 238 | 100 |

*The VOCs are Alpha, Delta, and Omicron; the VOI is Eta; the decreasing proportions were as follows: the 21K and 21L (Omicron) represent 53% (n = 126) of the dataset; the 21A, 21I and 21J (Delta) 31% (n = 74); 20A 9,7% (n = 23); 20B 3,3 % (n = 8); 20D 1,3% (n = 3); 21I (Alpha) 1,3% (n = 3) and 21D (Eta) 0,4% (n = 1). NA, not applicable

# Figure S1 Temporal overview of sequencing events at CRV-LFHVG. Each round colored dot in the main plot represents a sample sequenced retrospectively (blue) or prospectively (red), depending on the time elapsed from sampling to sequencing. Samples sequenced 14 days or more since the sampling day were considered retrospectively sequenced. Prospective sequencing was considered the run performed within 14 days since sampling. Black squares denote days when sequencing was performed in the lab. The vertical dotted line represents the time when CRV-LFHVG sequencing lab became fully operational (mid-October 2021). The upper-left boxplot shows the sample load per run, with the median value indicated inside the boxplot.

#
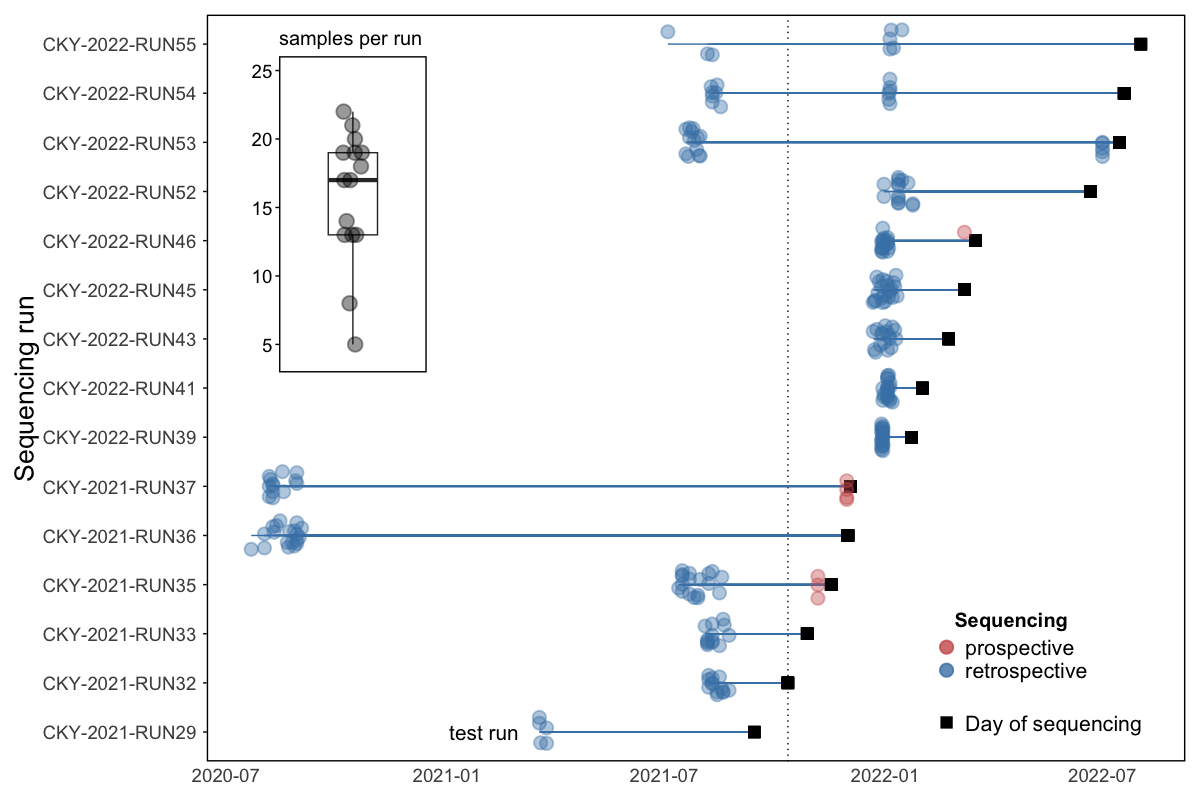


# Figure S2

**Example of nanopore sequencing laboratory minimal setup.**

Schematic representation of a SARS-CoV-2 nanopore next generation sequencing laboratory for setup in remote settings, as in CRV-LFHVG. To overcome troubleshooting and sustain uninterrupted activities, every piece of laboratory or sequencing equipment is available at least in duplicate. Created in BioRender. Duraffour, S. (2025)

**
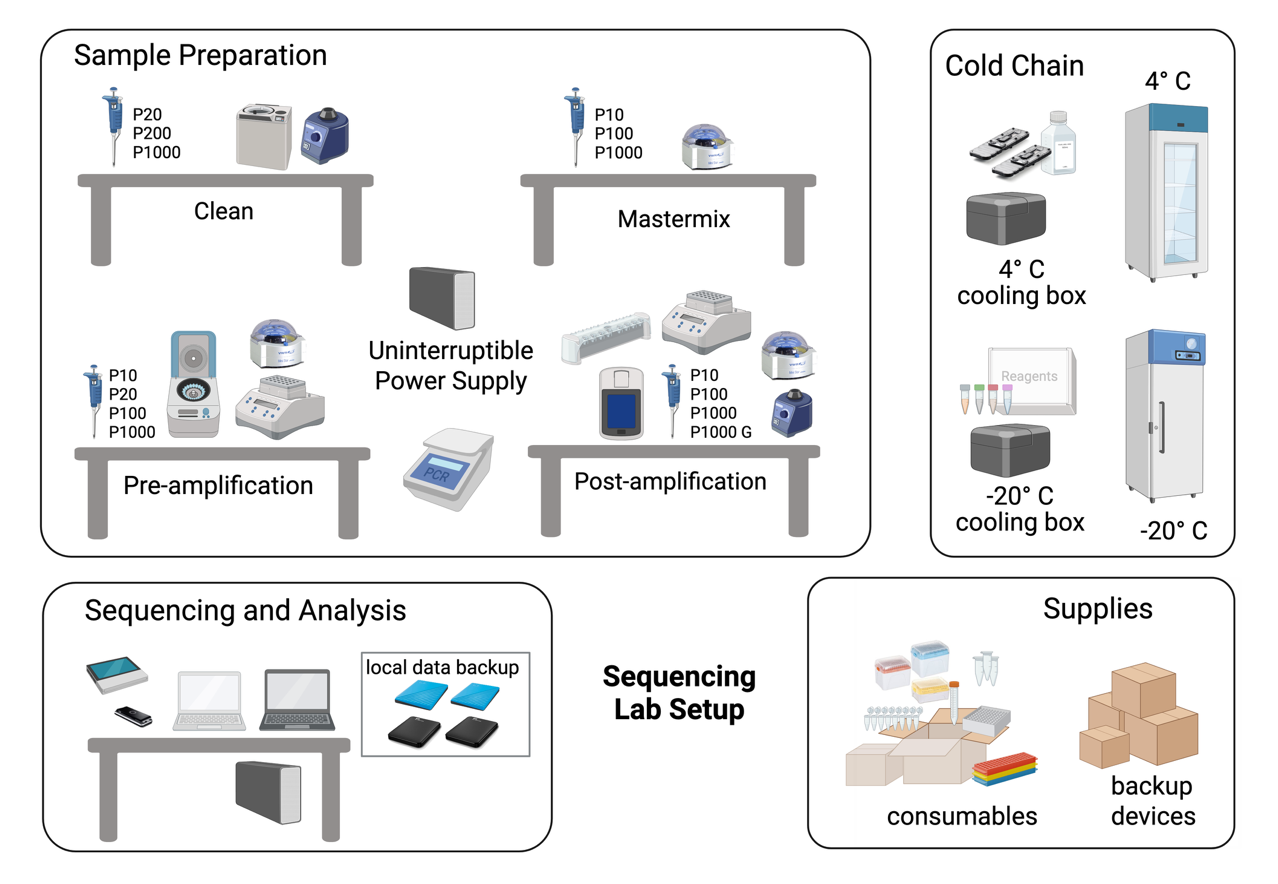
**

# File S1

**Metadata**

**Data Availability**

GISAID Identifier: EPI_SET_250318ve

DOI: https://doi.org/10.55876/gis8.250318ve

All genome sequences and associated metadata in this dataset are published in GISAID’s EpiCoV database. To view the contributors of each individual sequence with details such as accession number, Virus name, Collection, date, Originating Lab and Submitting Lab and the list of Authors, visit 10.55876/gis8.250107tk

**Data Snapshot**

EPI_SET_250318ve is composed of 10,621 individual genome sequences.

The collection dates range from 2019-12-31 to 2022-11-08;

Data were collected in 176 countries and territories.
